# A Canadian Perspective on Perioperative Systemic Therapy in Resectable Non-Small Cell Lung Cancer

**DOI:** 10.3390/curroncol33010020

**Published:** 2025-12-30

**Authors:** Saqib Raza Khan, Enxhi Kotrri, Daniel Breadner, Vijayananda Kundapur, Mita Manna

**Affiliations:** 1 Division of Medical Oncology, Department of Oncology, Schulich School of Medicine & Dentistry, Western University, London, ON N6A 3K7, Canada; saqib.khan@lhsc.on.ca (S.R.K.); daniel.breadner@lhsc.on.ca (D.B.); 2 Verspeeten Family Cancer Centre, London Health Sciences Centre, London, ON N6A 5W9, Canada; enxhi.kotrri@lhsc.on.ca; 3 Division of Radiation Oncology, Department of Oncology, Schulich School of Medicine & Dentistry, Western University, London, ON N6A 3K7, Canada; 4 Department of Radiation Oncology, Saskatchewan Cancer Agency, University of Saskatchewan, 20 Campus Drive, Saskatoon, SK S7N 5A2, Canada; vijayananda.kundapur@saskcancer.ca; 5 Department of Medical Oncology, Saskatchewan Cancer Agency, University of Saskatchewan, 20 Campus Drive, Saskatoon, SK S7N 5A2, Canada

**Keywords:** non-small cell lung cancer, resectable NSCLC, perioperative therapy, immunotherapy, targeted therapy

## Abstract

The management strategies in resectable non-small cell lung cancer (NSCLC) have changed over the last few years. Despite advancements in surgical techniques and conventional chemotherapy, patients with resectable NSCLC remained at high risk of future recurrence. Clinical trials have demonstrated improvements in response rates, pathological outcomes, and survival with the perioperative approach. Considering the findings of these landmark trials, there is a pressing need to contextualize and incorporate these global developments into the national practice framework. This review outlines key developments from recent clinical trials, with a focus on perioperative strategies in early-stage operable NSCLC from a Canadian perspective. We discuss the integration of checkpoint inhibitors in the perioperative setting for patients without actionable genomic alterations, adjuvant targeted therapies for EGFR and ALK mutant disease, and emerging tools such as ctDNA based minimal residual disease monitoring. The article also addresses the practical challenges of implementing these advances within the Canadian healthcare system, including systemic therapy approvals, barriers, and importance of multidisciplinary care to guide clinicians in optimizing patient outcomes.

## Simple Summary

Lung cancer is the leading cause of cancer death across the globe. The vast majority of cases are a type called non-small cell lung cancer, which includes common subtypes like adenocarcinoma and squamous cell carcinoma. Many patients remain at risk of cancer coming back even after being caught early and surgically removed. Previous studies have shown that adding chemotherapy after surgery helps patients live longer. Recently, major clinical trials have reported that treatments such as immunotherapy and targeted therapy before and/or after surgery (called perioperative therapy) can further improve patients’ chances of living longer without cancer coming back. This review summarizes recent clinical trial data and how they can be integrated into Canadian clinical practice.

## 1. Introduction

Lung cancer is the second most common cancer globally, and it is the leading cause of cancer-related mortality in both men and women [1]. In 2024, the Canadian Cancer Society reported lung cancer as the leading cancer, with an incidence of 32,100 and a mortality of 20,700 [2]. Non-small cell lung cancer (NSCLC) makes up most (80–85%) of the lung cancer cases, with adenocarcinoma being the most common pathology [1]. Most NSCLC cases present at advanced stages, with poor survival outcomes and a 5-year survival rate of less than 10% [3]. The survival rate in patients with early-stage NSCLC varies based on stage and presence of nodal disease, ranging from 33 to 65% [3,4]. The addition of adjuvant systemic treatment for early-stage NSCLC has been shown to improve survival outcomes by about 5% [5].

Management strategies in early-stage NSCLC have changed over the last few years with recent advancements in diagnostic techniques, molecular profiles, the use of immune checkpoint inhibitors (ICIs), and other targeted therapies. Furthermore, in addition to adjuvant strategies, numerous clinical trials have demonstrated efficacy and improved outcomes utilizing systemic treatment in the neoadjuvant and perioperative setting. This review outlines key developments from recent clinical trials, with a focus on perioperative strategies in early-stage operable NSCLC from a Canadian perspective.

## 2. Previous Treatment Landscape of Resectable NSCLC

Randomized controlled trials (RCTs) have demonstrated that adjuvant systemic chemotherapy reduces the risk of death, with trends towards improved survival [6,7,8,9,10,11]. The Lung Adjuvant Cisplatin Evaluation (LACE) performed a meta-analysis including 4584 patients which demonstrated that following surgery, cisplatin-based systemic chemotherapy improved 5-year OS by an absolute 5.4% with no apparent differences among other regimens, such as vinorelbine or etoposide, and an 11% reduction in death risk (HR: 0.89, *p* = 0.005) [5]. The benefit was mostly limited to stages II and III. In contrast, the Cancer and Leukemia Group B (CALGB) 9633 study compared adjuvant carboplatin-paclitaxel with observation in early-stage IB patients. The final updated 9-year follow-up showed a trend toward improved OS (HR = 0.80; 90% CI, 0.63–1.02; *p* = 0.062, one-tailed) and a 7% advantage in 8-year OS favoring adjuvant carboplatin-paclitaxel (51% vs. 44%; *p* = 0.087) [12]. A consistent OS benefit (HR: 0.77, 90% CI: 0.57–1.04; *p* = 0.079) was observed in patients with tumors of 4 cm or greater in size [12].

Based on the clinical trial findings, guidelines recommend adjuvant chemotherapy in patients with completely resected stage IIA and above and among selected patients of stage IB with one or more high-risk features, including tumor size (4 cm), poorly differentiated pathology, vascular invasion, visceral pleural involvement, wedge resection, unknown nodal status, and/or high standardized uptake value (SUV) on positron emission tomography (PET) scan [13,14,15]. Table S1 summarizes the adjuvant chemotherapy trials for patients with early-stage NSCLC.

## 3. Perioperative Assessment in Resectable NSCLC

### 3.1. Patient Selection and Comprehensive Staging

Appropriate patient selection and staging are essential for the management of resectable NSCLC. This includes a thorough history and physical examination, as well as imaging of the chest, abdomen, and pelvis with a Computed Tomography (CT) scan, a positron emission tomography (PET) scan, and CT or Magnetic Resonance Imaging (MRI) brain imaging if clinically symptomatic. For those with suspected mediastinal nodal involvement, endobronchial ultrasound-guided transbronchial needle aspiration (EBUS-TBNA) or endobronchial ultrasound-guided fine needle aspiration (EBUS-FNA) is recommended for assessing nodal stations [16,17,18].

### 3.2. Molecular and Genomic Alterations and Circulating Tumor DNA (ctDNA) Testing in Perioperative Assessment for Operable NSCLC

**Figure 1:**
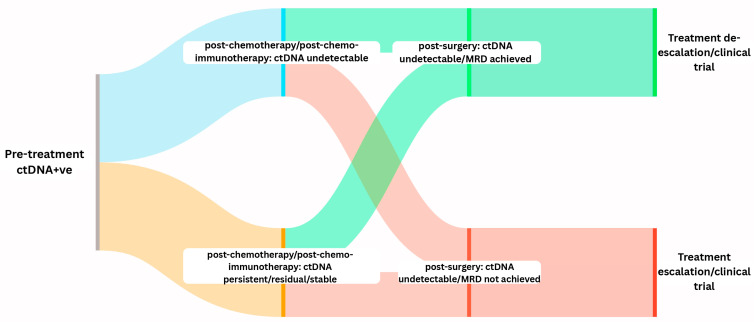
Hypothetical conceptual diagram illustrating serial ctDNA/MRD monitoring in resectable NSCLC, illustrating how serial ctDNA monitoring can risk-stratify NSCLC patients for MRD.

The era of precision oncology has gradually infiltrated early-stage non-small cell lung cancer (ES-NSCLC), modifying how physicians conduct diagnostic investigations, categorize risks, and plan therapies. This fundamental transformation involves comprehensive molecular profiling by Next-Generation Sequencing (NGS). While once recommended only in advanced settings, genomic molecular testing is now proving essential in guiding adjuvant and perioperative treatment strategies in operable NSCLC. The emergence of targeted therapies for EGFR mutations and ALK alterations, as well as PD-L1-directed therapies and their subsequent approvals in the adjuvant setting, marked a turning point that continues to redefine the standard of care in operable NSCLC. Moreover, the integration of circulating tumor DNA (ctDNA) and minimal residual disease (MRD) assessment is poised to revolutionize perioperative management in this cohort [19]. In comparison to tumor-agnostic methods, tumor-informed MRD assays exhibit superior sensitivity in tracking single-nucleotide variants (SNVs) and have demonstrated coherence and uniformity across global datasets comprising over 1600 patients [20,21,22]. Numerous studies confirm that residual ctDNA after surgery predicts recurrence, suggesting the need for escalation of treatment and the monitoring of ctDNA at different time intervals. On the other hand, clearance of ctDNA could support de-escalation of therapy and hence spare such patients from experiencing unnecessary toxicity [23]. Figure 1 represents a hypothetical conceptual diagram illustrating how serial ctDNA monitoring can risk-stratify NSCLC patients for MRD. Large, prospective, biomarker-driven clinical trials are needed to fully understand the utility of ctDNA in the context of clinical decision-making, to establish consensus on assay sensitivity thresholds, and to optimize perioperative treatment protocols in ES-NSCLC.

### 3.3. Role of Multidisciplinary Cancer Conference (MCC) Meetings

Multidisciplinary cancer conference (MCC) meetings play a crucial role in the comprehensive perioperative assessment and management of resectable NSCLC. Hung et al. reported that multidisciplinary meetings are an independent prognostic factor for improved survival in patients with lung cancer [24]. These organized forums bring together medical and radiation oncologists, thoracic surgeons, respirologists, pathologists, radiologists, nuclear medicine physicians, and interventional specialists, as well as input from clinical trial experts and social workers, to collaboratively construct a patient-centered treatment plan. Such rounds are crucial in managing complex or borderline resectable NSCLC cases, further ensuring consistent practice across the institution. Additionally, this approach not only promotes evidence-based practice but also enhances care coordination and reduces treatment delays.

## 4. Examining Immunotherapy Integration in Resectable NSCLC: Neoadjuvant, Perioperative and Adjuvant Approaches

Platinum-based systemic chemotherapy has been the mainstay of treatment for resectable NSCLC in the adjuvant setting for many years, providing a 5% absolute benefit in 5-year survival [5]. The integration of immunotherapy into the neoadjuvant, perioperative, or adjuvant setting demonstrated improvement in outcomes for patients with resectable NSCLC. Opting for an appropriate perioperative strategy is crucial, with informed decisions further guided by disease biology, nodal burden, and patient performance status.

### 4.1. Neoadjuvant Immunotherapy

The use of neoadjuvant immunotherapy in resectable NSCLC was initially examined in a feasibility study (Checkmate159) reported in 2018, where candidates with stage I-IIIA received two doses of perioperative nivolumab. Following this, many early phase I/II (e.g., NEOMUN, LCM3, NEOATSAR, NADIM) studies reported a major pathological response (MPR; defined as ≤10% viable tumor cells), pathological complete response (pCR), and disease-free survival (DFS) benefit with this approach [25,26,27,28,29,30,31,32]. The extended 5-year follow-up data from the NADIM phase II study of neoadjuvant nivolumab showed a PFS of 65% (95% CI: 49.4–76.9) and an OS of 69.3% (95% CI: 53.7–80.6) [33]. Following this, a phase II follow-up NADIM study enrolled 86 resectable stage IIIA-B NSCLC patients, randomized to receive three cycles of nivolumab plus carboplatin and paclitaxel versus chemotherapy alone. Patients in the experimental arm who achieved R0 resection received adjuvant 6 months of Nivolumab. The study reported a DFS of 67.2% vs. 40.9% (HR: 0.47; 95% CI: 0.25–0.88) and an OS of 85% vs. 63.6% (HR: 0.43; 95% CI: 0.19–0.98) at 24 months [34].

The landmark Checkmate-816 trial randomized 358 stage IB-IIIA resectable NSCLC patients with an ECOG-PS (Eastern Cooperative Oncology Group-Performance Status) of 0–1 and no known ALK or EGFR mutations to receive three cycles of nivolumab plus platinum-based chemotherapy or chemotherapy alone in the neoadjuvant setting [35]. The study reported a median event-free survival (EFS) that favored the combination arm (31.6 months vs. 20.8 months), with an HR of 0.63 (97.38% CI: 0.43–0.91, *p* = 0.005). Furthermore, the pCR was significantly improved with chemoimmunotherapy (24% vs. 2.2%; OR = 13.94 [3.49–55.75], *p* < 0.001). Long-term follow-up data are consistent with a sustained median EFS benefit (43.8 months vs. 18.4 months; HR: 0.66; 95%CI: 0.49–0.90) and a 13% improvement in OS (4-year OS rates of 71% vs. 58%) along with fewer grade 3 TRAEs (92.6% vs. 97.2%) [35,36].

### 4.2. Perioperative Immunotherapy

Checkmate-77T further strengthens the paradigm by adding one year of adjuvant nivolumab after neoadjuvant chemoimmunotherapy [37]. The trial investigated stage IIA-IIIB resectable NSCLC patients and assigned them either four cycles of neoadjuvant platinum-based chemotherapy and one year of adjuvant nivolumab or four cycles of neoadjuvant platinum-based chemotherapy alone and one year of adjuvant placebo. The 18-month EFS rate was 70.2% in the nivolumab group, compared to 50% in the chemotherapy-only group (HR: 0.58; 95% CI: 0.42–0.81; *p* < 0.001). The study reported an MPR rate of 35.4% and 12.1%, respectively (OR: 4.01; 95% CI: 2.48–6.49). Moreover, pCR also increased with the addition of neoadjuvant nivolumab (25.3% vs. 4.7%), with an OR of 6.64 (95% CI: 3.4–12.97). Wakelee et al. also investigated perioperative immunotherapy in a phase III (Keynote-671) study that randomized resectable stage II-IIIB NSCLC patients to receive pembrolizumab or placebo, administered every three weeks, along with four cycles of cisplatin-based systemic chemotherapy [38]. It was followed by surgery and then adjuvant pembrolizumab or placebo for 13 cycles, once every three weeks. The trial favored perioperative pembrolizumab, with an EFS of 62.4% versus 40.6% (HR: 0.58, CI: 0.46–0.72, *p* < 0.001), a pCR of 18.1% versus 4.0% (*p* < 0.001), and an improved OS of 80.9% vs. 77.6% (*p* = 0.02) [38].

The AEGEAN study investigated perioperative durvalumab in resectable stage II-IIIB NSCLC patients. The study randomized patients into two groups, one receiving a placebo and the other receiving durvalumab, with four cycles of neoadjuvant platinum-based systemic chemotherapy, followed by twelve cycles of either placebo or durvalumab in the adjuvant setting. The trial reported benefits in EFS and pCR with durvalumab. Although a consistent benefit was observed regardless of PD-L1 status, it was greater in patients with PD-L1 levels above 50% (HR: 0.60; 95% CI 0.33–1.38) [39]. A study from China, NEOTORCH, randomized 501 patients, with predominant squamous cell pathology (77%), to receive perioperative toripalimab [40]. The trial published data from a stage III resectable NSCLC cohort consisting of 404 patients, randomized to receive either three cycles of neoadjuvant toripalimab or placebo, followed by one cycle of adjuvant toripalimab or placebo, along with platinum-based systemic chemotherapy. This was followed by 13 cycles of toripalimab or placebo. The trial reported a median EFS which was not reached in the toripalimab group versus 15 months in the placebo group (HR: 0.40; 95% CI, 0.28–0.57; *p* < 0.001). There was a 40.2% (95% CI, 32.2–48.1%, *p* < 0.001) difference in the MPR between the two groups. Based on the significant EFS benefit, as well as improvements in pCR and MPR rates (Table 1), toripalimab has been approved in China for use in the perioperative setting [40].

### 4.3. Adjuvant Immunotherapy

There is also evidence for the addition of immunotherapy to chemotherapy in the adjuvant setting, in patients with resected NSCLC who did not receive neoadjuvant therapy. IMpower010 laid the foundation of this strategy and evaluated adjuvant atezolizumab (*n* = 507) versus best supportive care (*n* = 498) in stage IB-IIIA operable NSCLC after adjuvant cisplatin-based chemotherapy [41]. The trial investigated the stage IB-IIIA intention-to-treat (ITT) population, which included NSCLC patients with EGFR/ALK alterations, as well as the PD-L1-positive subpopulation (stages II-IIIA). Although the study reported a numerical DFS benefit in the ITT (HR: 0.81, 95% CI: 0.67–0.99; *p* = 0.040) population, a statistically significant and clinically meaningful DFS benefit was seen in patients with stage II-IIIA (HR: 0.79; 95% CI: 0.64–0.96; *p* = 0.020) and stage II-IIIA patients with PD-L1 1% or more (HR: 0.66; 95% CI: 0.50–0.88; *p* = 0.0039). The final DFS and second interim OS were presented at the ASCO (American Society of Clinical Oncology) annual meeting in 2024 [42]. The study reported a consistent DFS benefit with stratified HRs (95% CI) of 0.85 (0.71–1.01), 0.83 (0.69–1.00), and 0.70 (0.55–0.91) in the ITT (stage IB-IIIA), stage II-IIIA, and 1% or more PD-L1-positive stage II-IIIA patients, respectively. Additionally, the stratified HRs (95% CI) for OS were 0.97 (0.78–1.22), 0.94 (0.75–1.19), and 0.77 (0.56–1.06), respectively. The investigators also reported unstratified HRs (95% CI) in the stage II-IIIA PD-L1 ≥ 50% population (*n* = 229), which were 0.48 (0.32–0.72) for DFS and 0.47 (0.28–0.77) for OS, and in the stage II-IIIA PD-L1 TC ≥ 50% without EGFR/ALK alterations (*n* = 209) population, which were 0.49 (0.32 to 0.75) and 0.44 (0.26 to 0.74), respectively [42]. These extended results support the use of adjuvant atezolizumab in a selected cohort of patients with PD-L1-positive disease.

O’Brien et al. reported the second interim analysis of the multicenter multinational phase III KEYNOTE-091/PEARLS study, which randomized 1177 stage IB-IIIA resected NSCLC patients to receive adjuvant pembrolizumab or placebo once every three weeks for up to 18 cycles [43]. Patients also received adjuvant chemotherapy per local guidelines. The trial reported a survival benefit regardless of the PD-L1 expression level. Although the study revealed a median DFS of 53.6 months in the pembrolizumab group versus 42.0 in the placebo group (HR: 0.76; 95% CI: 0.63–0.91, *p* = 0.0014) in the ITT population, in patients with PD-L1 tumor proportion score (TPS) of 50% or more, the median DFS improvement was not statistically significant (HR: 0.82, *p* = 0.14) [43]. Similar statistically non-significant results for DFS benefit were observed in the extended third interim analysis in the subgroup of patients with PD-L1 TPS of 50% or more, with a median DFS of 67.0 months versus 47.6 months (*p* = 0.13), respectively.

The findings from the IMpower010 and Keynote091 trials reinforce the concept of using adjuvant immunotherapy in patients with resected NSCLC after adjuvant chemotherapy. Nonetheless, the heterogeneity in benefits observed in the PD-L1 TPS > 50% subgroup in KEYNOTE-091 contrasts with the more uniform benefit in IMpower010, prompting further investigations to refine predictive criteria and understand the biological differences across the trial population. Table 1 summarizes the key immunotherapy trials in the neoadjuvant, perioperative, and adjuvant setting for resectable NSCLC patients.

**Table 1:** Key phase III immunotherapy trials in the neoadjuvant, perioperative, and adjuvant setting in resectable NSCLC.

Study/Ref.	N	Study Setting (Neoadjuvant/ Perioperative or Adjuvant)	Immunotherapy Agent (+/− Chemotherapy)	Pathological Response	Comments	Health Canada- Approved (Yes/No)
Checkmate-816 [35,36]	358	Neoadjuvant	Nivolumab + platinum-based chemotherapy (vs. placebo + chemotherapy)	pCR = 24% vs. 2.2%	Phase III EFS = 43.8 months vs. 18.4 months (HR: 0.66; 95%CI: 0.49–0.90), 4-year OS rates of 71% vs. 58%.	Yes
Checkmate-77T [37]	461	Perioperative	Nivolumab + platinum-based chemotherapy (vs. placebo + chemotherapy)	MPR = 35.4% vs. 12.1%, pCR = 25.3% vs. 4.7%	Phase III 18-month EFS = was 70.2% vs. 50% (HR: 0.58; 95% CI: 0.42–0.81; *p* < 0.001).	No
Keynote-671 [38]	797	Perioperative	Pembrolizumab + cisplatin-based chemotherapy (vs. placebo + chemotherapy)	MPR = 30.2% vs. 11%,	Phase III EFS = 62.4% vs. 40.6% (HR: 0.58, CI: 0.46–0.72, *p* < 0.001), OS = 80.9% vs. 77.6%, *p* = 0.02.	Yes
AEGEAN [39]	802	Perioperative	Durvalumab + platinum-based chemotherapy (vs. placebo+ chemotherapy)	pCR = 17.2% vs. 4.3%	Phase III EFS = 73.4% vs. 64.5% and 63.3% vs. 52.4% at 12 and 24 months, respectively, HR 0.68 (95% CI, 0.53–0.88, *p* = 0.004).	No
RATIONALE-315 [44]	453	Perioperative	Tislelizumab + platinum-based chemotherapy (vs. placebo + chemotherapy)	MPR = 56% vs. 15%.	Phase III An EFS benefit was observed with Tislelizumab (HR: 0.56, 95% CI: 0.40–0.79, *p* = 0.0003) compared to the placebo.	No
IMpower 010 [41,42]	1005	Adjuvant	Atezolizumab (vs. BSC)	-	Phase III Unstratified HRs (95% CI) in stage II-IIIA PD-L1 ≥ 50% population were 0.48 (0.32–0.72) for DFS and 0.47 (0.28–0.77) for OS.	Yes
KEYNOTE 091/PEARLS [43]	1170	Adjuvant	Pembrolizumab	-	Phase III Median DFS of 53.6 vs. 42.0 months (HR: 0.76; 95% CI: 0.63–0.91, *p* = 0.0014), Subgroup with PD-L1 TPS of 50% or more, median DFS of 67.0 months versus 47.6 months (*p* = 0.13).	Yes

MPR: major pathological response; pCR: pathological complete response; DFS: disease-free survival; OS: overall survival; EFS: event-free survival; BSC: best supportive care; PD-L1: programmed death ligand 1; TPS: tumor proportion score; HR: hazard ratio; CI: confidence interval.

The collective evidence from neoadjuvant, perioperative, and adjuvant immunotherapy trials demonstrated immune checkpoint inhibitors as a transformative element of curative intent treatment for resectable NSCLC. Across multiple key phase III studies, perioperative immunotherapy consistently improves pathological response rates and event free survival with emerging overall survival benefits. For Canadian clinicians, neoadjuvant chemoimmunotherapy should now be considered a standard option for medically fit patients with stage II to IIA and selected stage IB operable NSCLC without AGA, provided they have good ECOG performance status and discussed in MCC meetings. Key phase III adjuvant immunotherapy trials, as discussed, highlight a need for Canadian physicians to test for PD-L1 status, as adjuvant immunotherapy remains relevant for patients who did not receive neoadjuvant treatment. Notably, patients with EGFR and ALK alterations should generally be excluded from the immunotherapy-based perioperative approach due to limited benefit and the availability of superior targeted options.

Many other clinical trials are evaluating the role of immunotherapy in the neoadjuvant, perioperative, and adjuvant settings. Table S2 summarizes the key ongoing trials.

## 5. Targeted Therapies for Resectable NSCLC with Actionable Genomic Alterations

Perioperative treatment approaches in patients with actionable genomic alteration (AGA) require careful consideration. Although immunotherapy in the perioperative setting has become standard for the majority of resectable NSCLC patients, as discussed above, its applicability does not extend to patients harboring certain actionable mutations. Results from many clinical trials in advanced settings have shown limited efficacy of immunotherapy in patients whose cancers have EGFR, ALK, ROS1, and RET alterations. Moreover, most perioperative trials testing ICIs either excluded such patients or included a small subset of them. Consequently, the role of targeted therapies in the context of operable NSCLC has recently gained attention following the landmark trials of tyrosine-kinase inhibitors (TKIs) (ADAURA, ALINA), which led to FDA approval for osimertinib and alectinib in the adjuvant treatment of EGFR- and ALK-positive disease, respectively.

### 5.1. EGFR-Directed Therapies

Early studies evaluating perioperative TKI in resectable NSCLC reported modest results. Small phase II/III studies (NCIC CTG BR19, IMPACT, and CTONG1104) evaluated gefitinib and reported an ORR range from 11 to 50% without OS benefit [45,46,47,48,49,50]. A relatively higher ORR of >50% was seen with erlotinib in this cohort, with the 5-year OS rate reaching up to 86% (EMERGING-CTONG 1103, RADIANT and SELECT) [51,52,53,54,55,56,57]. Likewise, neoadjuvant afatinib reported an ORR of 70% with a modest MPR (9.1%) and pCR (3.0%), emphasizing the challenge of achieving deep pathological responses with brief cytostatic TKI treatment [58].

In the growing evidence, the third-generation TKI osimertinib has firmly established itself as a front-runner in the treatment of resectable EGFRm-positive NSCLC. The landmark ADAURA trial redefined the role of adjuvant osimertinib as a standard-of-care treatment in the resected stage IB-IIIA EGFR-mutant NSCLC. This largest international phase III clinical trial randomized 682 patients with exon 19 deletions (Ex19del) and exon 21 codon p.Leu858Arg (L858R) point mutations to receive either osimertinib or placebo for three years after surgery, with or without prior systemic chemotherapy. The study demonstrated a substantial 83% reduction in the risk of recurrence of stage II-IIIA disease (HR: 0.17; 99.06% CI: 0.11–0.26; *p* < 0.001) and 80% reduction across the entire (stage IB-IIIA) cohort (HR: 0.20; 99.12% CI, 0.14 to 0.30; *p* < 0.001). The benefits were consistent across all subgroups, regardless of receipt of adjuvant therapy, and in preventing central nervous system (CNS) and distant recurrences [59]. Updated survival analysis in 2023 confirmed OS benefit with a five-year OS rate of 85% in the osimertinib arm versus 73% with placebo (HR: 0.49; 95% CI: 0.33–0.73; *p* < 0.001) [60]. Previous studies (EVAN and ADJUVANT/CTONG1104) designed the 2-year duration of adjuvant TKI [61]; however, ADAURA considered 3 years of adjuvant osimertinib. The exploratory analysis of ADAURA revealed that patients who received adjuvant TKI for 18 months or more benefited in terms of DFS (HR: 0.38, 95% CI: 0.22–0.66), compared to those with less than 18 months of TKI exposure [62].

Blakely CM et al. evaluated the role of neoadjuvant osimertinib for a median of 56 days in stage I-IIIA EGFRm-positive NSCLC. The trial reported an ORR of 52%, MPR of 14.8% (95% CI: 4.2–33.7), and a median DFS of 40.9 months [63]. Although the trial did not meet its primary endpoint for MPR rate, the study showed an acceptable alternative for a likely subset of patients who cannot undergo neoadjuvant chemotherapy, with manageable safety and without surgical delays. Similarly, the NEOS study reported an ORR of 71.1% (95% CI: 55.2–83.0) with neoadjuvant osimertinib. Furthermore, the exploratory analysis by the authors has implicated co-mutations, such as loss-of-function of RBM10, as a possible resistance mechanism that mitigates the effect of TKI in the neoadjuvant setting [64]. Reports also suggested a poor DFS benefit with adjuvant TKI in patients with underlying nucleotide polymorphism mutations in UBXN11 [61].

Following this, data from NeoADAURA presented in 2025 demonstrated statistically significant improvement in MPR rates with neoadjuvant osimertinib with or without combination chemotherapy (26% and 25%, respectively), compared to the placebo plus chemotherapy arm (2%) in resectable stage II-IIIB EGFR-mutated NSCLC (*p* < 0.0001) [65].

### 5.2. ALK-Directed Therapies

Evidence of ALK-directed perioperative targeted therapies is also becoming prominent in resectable NSCLC. Small studies reported an R0 resection rate up to 90% with neoadjuvant crizotinib or ceritinib [66,67] and an MPR rate of 57% (95% CI: 18–90) [67]. The largest global phase III ALINA trial randomized 257 patients with resected stage IB-IIIA to receive either adjuvant alectinib for 24 months or three months of platinum-based chemotherapy. At 2 years, 93.6% in the alectinib arm versus 63.7% in the chemotherapy arm remain disease-free (HR: 0.24; 95% CI: 0.13–0.43; *p* < 0.001). Alectinib was also associated with clinically meaningful benefit in terms of CNS disease-free survival (HR: 0.22; 95% CI: 0.08–0.58) [68]. Both ADAURA and ALINA trials have reshaped the management landscape for adjuvant treatment in resected oncogenic-driven NSCLC, reducing the risk of disease recurrence and empowering CNS protection. Recently, the ALNEO (GOIRC-01-2020-ML42316) data, presented at ASCO-2025, included 33 patients who received neoadjuvant alectinib for 8 weeks (2 cycles) and adjuvant alectinib for 96 weeks (24 cycles). The study reported an MPR of 46% (90% CI: 31–61%), a pCR rate of 12% (95% CI: 3–28%), and an overall OR of 67%. Moreover, the median interval from surgery was 5.1 weeks (IQR, 3.6–6.0 weeks), and the EFS and OS were not reached [69]. With these results, alectinib may be a feasible option in the perioperative setting; however, long-term survival results and more mature randomized phase III clinical trials are required. Several other clinical trials (NCT04302025, NCT06755684, NCT01091376, NCT04197076, NCT06282536, NCT06682884, NCT06893354, NCT05765877, NCT05361564, NCT05380024, and NCT06779539) are currently investigating the efficacy of targeted treatment in driver mutation-positive resectable NSCLC in the neoadjuvant, perioperative and adjuvant settings. Table 2 summarizes the key phase III trials examining targeted therapies in resectable NSCLC.

The above studies firmly consolidated adjuvant osimertinib and alectinib as the current standard of care for resected EGFR- and ALK-positive NSCLC cohorts, respectively. For Canadian physicians, these findings strengthen the need for routine and timely molecular profiling, ideally prior to the initiation of perioperative treatment, in all patients with resectable NSCLC. Adjuvant chemotherapy remains appropriate for the selected cohort (resected stage IB-IIIA); however, targeted therapy should not be withheld based on chemotherapy use, given the consistent benefit across trials. Neoadjuvant TKI remains investigational; hence, the approach needs further mature data. Several aspects, such as the optimal duration of targeted treatment, the integration of chemotherapy, and the role of ctDNA and MRD monitoring, highlight the significance of continued clinical trial enrolment and real-world data collection in Canada.

## 6. Role of Radiation in the Treatment of Resectable NSCLC

### 6.1. Role of Radiation in Medically Unresectable Early-Stage NSCLC

Surgery remains the standard of care for resectable early-stage (stages I-II, N0) NSCLC, and definitive radiation is reserved for patients unable to undergo a resection. The Canadian MISSILE phase II prospective trial studied rates of pCR in patients with stage T1-2 N0 M0 NSCLC who underwent stereotactic body radiation therapy (SBRT), followed by surgery ten weeks later [71]. The update of long-term outcomes for these patients showed a pCR of 60% and MPR of 63% [72]. The 5-year OS was 66.7% (95% CI, 48.8–79.5%). Overall, the MISSILE study showed that the combination of neoadjuvant SBRT and surgery is safe and feasible, with long-term outcomes similar to those of surgery alone. Another phase II trial comparing neoadjuvant durvalumab with or without SBRT in early-stage NSCLC showed a significantly higher MPR with dual therapy (53% vs. 7%, *p* < 0.0001) [73], though the radiation dose used was lower than standard ablative doses [74], as radiation was intended for immune stimulation rather than for local control. The relevant studies are summarized in Table S3.

### 6.2. Neoadjuvant Radiation for Locally Advanced Resectable NSCLC (Non-Pancoast Tumors)

**Figure 2:**
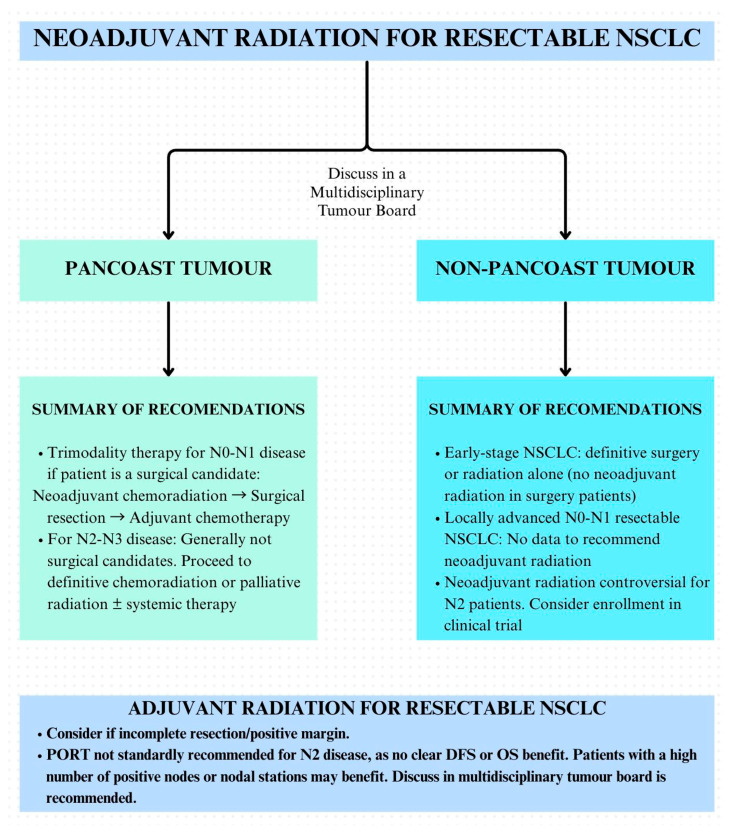
Summary of recommendations for the use of neoadjuvant and adjuvant radiation in resectable NSCLC (N: nodal disease; NSCLC: non-small cell lung cancer; PORT: post-operative radiotherapy; DFS: disease-free survival; OS: overall survival).

In patients with locally advanced NSCLC who may be surgical candidates, the role of neoadjuvant radiation remains unclear, and these cases should be discussed in an MCC to assess resectability up-front and guide treatment [75]. For resectable locally advanced N0–N1 disease, neoadjuvant systemic therapy may be considered; however, there is no prospective evidence for the use of neoadjuvant radiation in this setting. Patients with N2 NSCLC planned for definitive treatment either undergo definitive concurrent chemoradiation or surgical resection combined with perioperative systemic therapy [75,76]. In resectable N2 disease, several phase III trials have shown that while adding radiation to induction chemotherapy can improve mediastinal nodal clearance, pathologic response and local control, these benefits have not translated into consistent gains in progression-free or overall survival compared with neoadjuvant chemotherapy alone or definitive chemoradiation [77,78,79,80]. One of these studies, the landmark INT 0139 trial, suggested an improved median survival for patients who underwent lobectomy compared to definitive chemoradiation (33.6 vs. 21.7 months, *p* = 0.002). However, caution is warranted when interpreting these results as this was on exploratory analyses [81,82,83,84,85]. Some of the ongoing clinical trials are outlined in Tables S3 and S4. The summary of recommendations for neoadjuvant radiation for resectable NSCLCs is highlighted in Figure 2.

### 6.3. Neoadjuvant Radiation for Resectable Superior Sulcus Tumor

Pancoast or superior sulcus tumors (SST) are rare NSCLCs of the lung apex, classified as at least T3 or T4 depending on local invasion [86]. Once deemed incurable, these tumors became treatable following early reports showing that preoperative radiation could facilitate surgical resection [87]. Subsequent studies established trimodality therapy, consisting of neoadjuvant chemoradiation followed by surgery and adjuvant chemotherapy, as the standard of care. The 2007 SWOG 9416 phase II trial showed that neoadjuvant chemoradiation followed by surgery for superior sulcus N0-N1 NSCLC achieved a 94% complete resection rate, 69% pathologic complete response, and 5-year OS of 54% in patients with R0 resection, with no differences seen between T3 and T4 tumors [88]. Similarly, the JCOGT 9806 phase II trial and other systematic reviews support this approach [89,90,91]. Overall, the Canadian standard of care for Pancoast tumors with N0-N1 disease remains trimodality therapy. The relevant studies are summarized in Table S3.

### 6.4. Role of Adjuvant Radiation in the Treatment of Resectable

The role of postoperative radiotherapy (PORT) in NSCLC remains controversial. Early meta-analyses suggested that PORT may reduce survival in completely resected NSCLC, with a 7% absolute decrease reported in one analysis of nine randomized trials (HR 1.21) [92] and a 5% decrease at 2 years in a 2016 update of eleven trials [93]; however, this effect was no longer significant after reclassification using the updated TNM system (HR 0.87, 95% CI 0.72–1.04, *p* = 0.12). Retrospective studies suggested a survival benefit of PORT in patients with resected pN2 NSCLC, including analyses from the SEER database of 7465 patients (HR 0.855; 95% CI 0.762–0.959; *p* = 0.0077) [94] and the NCDB analysis of 4483 patients (HR 0.888; 95% CI 0.798–0.988; *p* < 0.014) [95]. Further reviews and meta-analyses have identified a potential survival and local control benefit of PORT in resected pN2 NSCLC, particularly when delivered using modern linear accelerator-based techniques [96,97]. Despite these findings, phase III randomized controlled trials failed to demonstrate a statistically significant survival benefit for PORT in completely resected stage III pN2 NSCLC [98,99].

The current Canadian standard is that PORT should be considered in the setting of an incomplete resection with a positive margin, following discussion within a multidisciplinary team. Radiation is not generally recommended for patients with completely resected N2 NSCLC, especially when neoadjuvant or adjuvant systemic therapy has been delivered. However, it should be noted that practice variations may exist across institutions. Novel approaches integration radiation with immune checkpoint inhibitors in early-stage resectable NSCLC are actively evolving; however, the approach should be restricted to clinical trials, as there is insufficient data to support this strategy in Canadian clinical practice. Table S4 summarizes the key ongoing trials investigating SBRT with immune checkpoint inhibitors for early-stage NSCLC. Canadian clinicians should continue to rely on MCC evaluation to determine resectability and treatment sequencing with neoadjuvant chemotherapy or chemoimmunotherapy favored in resectable NSCLC and definitive chemoradiation therapy reserved unresectable or surgically unfit patients. This is summarized in Figure 2.

## 7. Perspectives and Ongoing Advances

**Figure 3:**
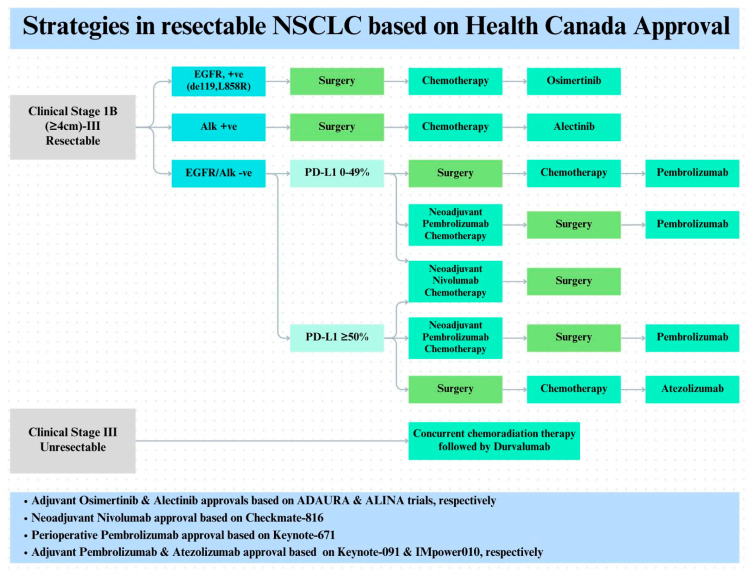
Treatment approach in resectable NSCLC based on Health Canada approval to date. Adjuvant osimertinib and alectinib approvals are based on ADAURA and ALINA trails, respectively. Adjuvant pembrolizumab and atezolizumab approvals are based on Keynote091 and IMpower-010, respectively. Neoadjuvant nivolumab approval is based on CheckMate-816 and perioperative pembrolizumab approval is based on Keynote-671 study.

Perioperative management in resectable NSCLC is now standard in Canadian practice, with data supported by landmark trials as discussed above, including CheckMate 816, KEYNOTE-671, IMpower010, KEYNOTE-091, ADAURA, and ALINA [100] (Figure 3). The implementation is still uneven across provinces and territories. Moreover, many other clinical trials are underway. Timelines for public funding of novel therapies varies across Canada. For example, neoadjuvant nivolumab as per CheckMate 816 is publicly covered in Ontario. However, adjuvant nivolumab for eligible patients requires compassionate access. Likewise, access to such agents can be hindered by local formulary approval within regional cancer agencies.

The future perspective in the perioperative management of operable NSCLC cohorts in Canadian healthcare practice will be defined by a shift towards precision, biomarker-guided treatment, and adaptive risk approaches that utilize molecular diagnostics, ctDNA dynamic monitoring, MRD assessment, and optimizing the sequence of systemic therapies to maximize cure while minimizing unnecessary side effects. In Canada, there are currently limitations to ctDNA testing, as it is not readily available nor funded in all provinces. Additionally, turnaround times of molecular results also differ across the country and in different institutions. Centralized laboratories in major academic institutions may offer extended NGS panels; however, rural and remote regions often rely on send-out tests, causing delays that significantly affect perioperative planning. As more mature data accumulate, ctDNA testing and MRD monitoring are likely to transition from research to routine clinical practice, empowering Canadian physicians to refine prognostication and personalize perioperative management dynamically. Tumor-informed ctDNA techniques have shown prognostic importance [20], with a positive assay postoperatively predicting early disease recurrence; however, on the other hand, clearance may justify de-escalation of adjuvant treatment. Following this approach, physicians may intervene pre-emptively and modify or intensify treatment before radiological disease relapse and even consider active surveillance for low-risk patients, thereby sparing them from extended adjuvant treatment.

Immune checkpoint inhibitors have garnered substantial evidence and have become the backbone of perioperative treatment in operable NSCLC patients without AGA, offering consistent benefits in the context of pCR, MPR, and survival. The scientific rationale behind the considerable efficacy of ICIs in the perioperative setting may reside in the presence of intact tumor antigens before resection, which enables effective T cell priming and the development of durable immune responses. As perioperative ICI use expands, future research will refine optimal combinations, treatment durations, and the role of ICI doublets targeting PD-1/PD-L1, CTLA-4, TIGIT, LAG-3, and other pathways in the perioperative setting. Moreover, other biomarker-based treatment combinations, such as TROP2-targeted (e.g., sacituzumab deruxtecan, datopotamab deruxtecan) or CEACAM5-targeted (e.g., tusamitamab ravtansine) antibody–drug conjugates (ADCs) in combination with ICIs, should be explored in large prospective clinical trials in the perioperative setting, as these ADCs now have an established role in subsequent-line advanced NSCLC with clinically meaningful outcomes [101,102,103]. Studies focusing on biomarker expression levels, sequencing of perioperative treatment, integration of ctDNA, and dynamic MRD monitoring could change the future of how we treat operable NSCLC patients at the current time.

For patients with a positive oncogenic driver mutation, determining the most effective therapy remains an unmet need, particularly as data on TKIs in the perioperative setting expands. The divergence between the cytotoxic effect of chemotherapy, which leads to cancer cell lysis, and the immunogenic cell death and the cytostatic behavior of the TKI, which halts proliferation without deep tumor eradication, has practical implications for perioperative strategies. While targeted drugs have demonstrated impressive radiological response rates in advanced NSCLC, perioperative trials in resectable disease often report modest MPR and pCR rates due to the limited duration of neoadjuvant TKI exposure and cytostatic nature of these drugs. Although the adjuvant long-term exposure to TKIs, as per ADAURA and ALINA, showed a survival benefit, with 90% of patients remaining disease-free at 24 months, parallel trials of neoadjuvant osimertinib (NeoADAURA) and alectinib (ALNEO) reported a modest pCR rate of 9–12% and an acceptable MPR despite a small sample size. One arm of NeoADAURA contained concurrent neoadjuvant chemotherapy, and in that arm, the risk of early failure was numerically less compared to osimertinib or chemotherapy alone. Within a short neoadjuvant window, it remains a challenge to achieve complete clearance with a TKI alone.

Current standards do not favor routine radiation therapy in neoadjuvant, perioperative, or adjuvant settings for resectable NSCLC. Adjuvant radiation (PORT) is a considerable option for patients with incomplete resection/positive margins and can be considered for individual patients after MCC discussion. Access to timely radiation therapy in Canada is, however, a major concern, especially outside major urban centers.

As detailed above, clinical decision pathway for resectable NSCLC in Canadian practice is evolving. For EGFR (exon 19 del/L858R mutation) and ALK alteration, adjuvant osimertinib and alectinib, respectively, are standard, while immunotherapy is generally avoided. For AGA-negative stage IB-IIIA NSCLC patients, perioperative immunotherapy (neoadjuvant chemoimmunotherapy followed by adjuvant immunotherapy) is preferred, though neoadjuvant-only or adjuvant-only strategies are considerable alternatives based on patient preference and ECOG performance status. CtDNA-based MRD monitoring holds promise for refining risk stratification, guiding adjuvant therapy de-escalation in MRD-negative patients, and warranting early salvage intervention upon molecular disease recurrence.

Canadian healthcare system considerations and regulatory approvals will significantly shape the practical application and execution of these visionary approaches [104,105]. Discrepancies in provincial funding and timelines for the approval of innovative therapies, ctDNA testing, and comprehensive molecular profiling may contribute to inequities in access, impacting the consistency of perioperative care across Canada. Furthermore, indigenous, rural, and other remote population groups often face barriers to timely access to specialist physicians. Finally, multidisciplinary tumor rounds are imperative to the care and management of these patients and should be evaluated through a coordinated approach involving thoracic surgery, radiology, respirology, social work, medical and radiation oncology, and clinical trial teams. Although MCC is an integral aspect of decision-making in resectable NSCLC, its implementation faces real-world logistic challenges across Canada’s diverse healthcare geography. Moreover, care is often split between different organizations such as regional health authorities, private clinics, provincial cancer agencies, etc. Lack of integrated electronic medical records (EMRs) across these entities results in patient data not being easily accessible to all team members during MCC rounds, leading to incomplete discussions. Lack of dedicated and well-equipped rooms and poor audio-visual technology hinder effective visual participation for remote team members, making true collaboration difficult. The importance of these MCC rounds was recently emphasized in a Canadian study presented at World Conference on Lung Cancer (WCLC) 2025 [104].

## 8. Conclusions

The healthcare practice in Canada for the perioperative management of resectable NSCLC follows the global guidelines. With growing evidence, it will further incorporate dynamic molecular monitoring, rational sequencing of systemic treatments, and individualized patient selection, guided by ctDNA/MRD, resistance biology, and tumor immunology. Embedding these advancements into the Canadian healthcare system, with a focus on equitable access, will be crucial to ensuring that perioperative innovations translate into consistent and durable improvements in cure rates and quality of life for patients with resectable NSCLC.

## Figures and Tables

**Table 2:** Key phase III trials examining targeted therapies in resectable NSCLC.

Study/Ref.	N	Study Setting (Neoadjuvant/ Perioperative/ Adjuvant)	Target	Treatment	Brief Description	Health Canada- Approved (Yes/No)
NCIC CTG BR19 study [47]	503	Adjuvant	EGFR	Gefitinib vs. placebo	Phase III, Resected stage IB-IIIA, DFS = 4.2 years vs. NR (HR: 1.22; 95% CI: 0.93–1.61; *p* = 0.15), OS = 5.1 years vs. NR (HR: 1.24; 95% CI: 0.94–1.64; *p* = 0.14)	No
IMPACT [48]	234	Adjuvant	EGFR	Gefitinib vs. cisplatin + vinorelbine	Phase III, Resected II-IIIA, 5-year OS rate = 78% vs. 74.6%	No
ADJUVANT/CTONG1104 [49,50]	483	Adjuvant	EGFR	Gefitinib vs. cisplatin + vinorelbine	Phase III, Resected II-IIIA, DFS = 28.7 vs. 18.0 months (HR: 0.60, 95% CI 0.42–0.87; *p* = 0.0054), 5-year OS rate = 53.2% vs. 51.2% (*p* = 0.784)	No
RADIANT [56]	973	Adjuvant	EGFR	Erlotinib	Phase III, Resected stage IB-IIIA, Median DFS in EGFRm-positive subgroup = 46.4 vs. 28.5 months (HR: 0.61; 95% CI, 0.38- 0.98; *p* = 0.039)	No
EVIDENCE [70]	232	Adjuvant	EGFR	Icotinib vs. platinum-based chemotherapy	Phase III, Resected stage II-IIIA, Median DFS = 47 vs. 22.1 months (HR: 0.36; 95% CI: 0.24–0.55; *p* < 0.0001)	No
NeoADAURA [65]	358	Neoadjuvant	EGFR	Osimertinib + chemotherapy vs. osimertinib monotherapy vs. placebo + chemotherapy	Phase III, Clinical stage II-IIIB, MPR rates = 26%, 25%, and 2%, respectively (*p* < 0.0001), pCR rates are 4%, 9%, and 0%, respectively	No
ADAURA [59,60]	682	Adjuvant	EGFR	Osimertinib vs. placebo	Phase III, Resected stage IB-IIIA, DFS at 24 months = 90% vs. 44% (HR: 0.17; 99.06% CI: 0.11–0.26; *p* < 0.001), 5-year OS = 85% vs. 73% (HR: 0.49; 95% CI: 0.33–0.73; *p* < 0.001)	Yes
ALINA [68]	257	Adjuvant	ALK	Alectinib vs. platinum-based chemotherapy	Phase III, Resected stage IB-IIIA. At 2 years, patients who remain disease-free are 93.6% vs. 63.7%, respectively (HR: 0.24; 95% CI: 0.13–0.43; *p* < 0.001)	Yes

MPR: major pathological response; pCR: pathological complete response; HR: hazard ratio; CI: confidence interval; OR: odds ratio; PFS: progression-free survival; OS: overall survival; DFS: disease-free survival; ORR: objective response rate; EGFR: epidermal growth factor receptor; ALK: anaplastic lymphoma kinase.

## Data Availability

No new data were created or analyzed in this study.

## Supplementary Materials

The following supporting information can be downloaded at https://www.mdpi.com/article/10.3390/curroncol33010020/s1: Table S1: Historical systemic chemotherapy trials in early-stage resectable NSCLC; Table S2: Key ongoing clinical trials utilizing immunotherapy in resectable NSCLC; Table S3: Summary of the phase II and III trials analyzing radiation for resectable NSCLC; Table S4: Key ongoing clinical trials utilizing radiation in resectable NSCLC.

## Abbreviations

The following abbreviations are used in this manuscript:

NSCLC	Non-small cell lung cancer
ICI	Immune checkpoint inhibitors
RCT	Randomized controlled trials
ctDNA	Circulating tumor DNA
MRD	Minimal residual disease
MCC	Multidisciplinary cancer conference
