# Supplementary material for: A Canadian Perspective on Perioperative Systemic Therapy in Resectable Non-Small Cell Lung Cancer
Source: Curr Oncol. 2025 Dec 30;33(1):20. doi: 10.3390/curroncol33010020 (PMC12840373; doi:10.3390/curroncol33010020)
Supplement: Supplementary file 1 [file curroncol-33-00020-s001.zip › Supplementary File S1 (Table S1).pdf]

Table S1. Historical systemic chemotherapy trials in early-stage resectable NSCLC.

Trial	Study Design	Study Size (n) & Disease Stage	Systemic Chemotherapy	Comments	Ref.
ALPI	Multicentre, Phase III, RCT.	n= 1209<br>Stage I, II, IIIA	MVP regimen; Mitomycin C, vindesine, and cisplatin, once every 3 weeks for three cycles.	Median follow-up 64.5 months.<br><br>No difference in OS (HR = 0.96, 95% CI = 0.81 to 1.13; P = 0.589) and PFS (HR = 0.89, 95% CI 0.76 to 1.03; P =0.128) between the chemotherapy and observation arm.	[6]
BLT	Multicentre, Phase III, RCT	n= 381<br>Stage I, II, IIIA-C.	Regimens (once every three weeks for three cycles) as per the physician's choice, including;<br><br>MIC: cisplatin, mitomycin, ifosfamide<br><br>MVP: cisplatin, mitomycin, vinblastine.<br><br>CV: cisplatin, vindesine.<br><br>NP: cisplatin, vinorelbine.	Median follow-up was 34.6 months.<br><br>No difference in OS (HR: 1.02, 95% CI: 0.77–1.35, P = 0.90) was observed between patients with or without chemotherapy.	[7]
IALT	Multicentre, Phase III, RCT	n= 1867<br>Stage I, II, IIIA-C.	Cisplatin with either etoposide, vinorelbine, vinblastine, or vindesine in the	Median follow-up was 56 months.<br><br>Significant OS (44.5% vs. 40.4%, HR: 0.86,	[9]

			adjuvant setting.	95% CI: 0.76-0.98, P < 0.03) & DFS (39.4% vs. 34.3%, HR: 0.83, 95% CI: 0.74-0.94, P < 0.003) benefit was observed with chemotherapy compared to observation alone.	
JBR.10	Multicentre, Phase III, RCT	n= 482<br>Stage IB, II	Cisplatin plus vinorelbine in the adjuvant setting for a total of four cycles.	Median follow-up was 9.3 years in the updated analysis.<br><br>Significant OS (94 vs. 73 months; HR: 0.69; P = 0.04) and RFS (not reached vs. 47 months,<br>HR: 0.60; P < 0.001) was observed with chemotherapy versus observation.<br><br>The five-year survival rates were 69%<br>and 54%, respectively (P = 0.03). No benefit was seen in stage IB patients at 9-year follow-up.	[10]
ANITA	Multicentre, Phase III, RCT	n= 840<br>Stage IB, II, III.	Cisplatin plus vinorelbine in the adjuvant setting.	Median follow-up was 76 months.<br><br>Median OS was 65.7 months versus 43.7 months (95% CI: 47.9–88.5) in the chemotherapy versus observation arm; an 8.6% absolute increase in OS (p = 0.02) at 5	[11]

				years.	
LACE	Meta-analysis, pooled from five trials.	n= 4,584<br>Stage I, II, III	Cisplatin-based chemotherapy; associated drugs include vinorelbine, etoposide or vinca alkaloid, or others.	Median follow-up was 5.2 years.<br><br>5-year absolute benefit of 5.4% from the chemotherapy (Pooled HR: 0.89 (95% CI, 0.82 to 0.96; P = 0.005)	[5]
CALGB 9633	Multicentre, Phase III, RCT	n= 344<br>Stage IB	Carboplatin plus paclitaxel, once every three weeks for a total of four cycles.	The median follow-up was 9 years in the final overall survival (OS) data.<br><br>The initial 3-year survival data showed a significant OS benefit with chemotherapy compared to observation (80% vs. 73%, P = 0.02).<br><br>Long-term follow-up showed no significant advantage; however, a consistent OS benefit (HR: 0.77, 90% CI: 0.57-1.04; p = 0.079) was observed in patients with a tumor ≥4 cm.	[12]
TREAT	Multicentre, Phase II, RCT	n= 132<br>Stage IB, II, IIIA	Cisplatin plus vinorelbine or cisplatin plus pemetrexed; four cycles, once every three weeks.	Comparable 3-year survival rates (75% versus 77%, p = 0.858) were observed in both arms with adjuvant chemotherapy.	[14]

JLCRG	Multicentre, Phase III, RCT.	n= 979 stage I (T1N0M0 or T2N0M0) adenocarcinoma lung	Postoperatively, uracil–tegafur (250 mg tegafur per square meter of body surface area per day) was administered orally in the form of 100-mg capsules (containing 100 mg tegafur and 224 mg uracil) twice daily, before meals, for 2 years.	The median follow-up was 72 months in the uracil–tegafur group and 73 months in the control group.<br><br>The difference in OS between the two groups was statistically significant in favour of the uracil–tegafur group.<br><br>The T2N0 subset received the most benefit, with a 5-year overall survival rate of 85% compared to 74% in the control group ($p = 0.005$).	[15]

ALP: Adjuvant Lung Project Italy, BLT: Big Lung Trial, RCT: Randomized controlled trial, IALT: International Adjuvant Lung Cancer Trial, ANITA: Adjuvant Navelbine International Trialist Association, LACE: Lung Adjuvant Cisplatin Evaluation, CALGB: Cancer and Leukemia Group B, HR: Hazard ratio, JLCRG: Japan Lung Cancer Research Group, OS: Overall survival, PFS: Progression-free survival, RFS: Relapse-free survival.
