# Supplementary material for: A Canadian Perspective on Perioperative Systemic Therapy in Resectable Non-Small Cell Lung Cancer
Source: Curr Oncol. 2025 Dec 30;33(1):20. doi: 10.3390/curroncol33010020 (PMC12840373; doi:10.3390/curroncol33010020)
Supplement: Supplementary file 1 [file curroncol-33-00020-s001.zip › Supplementary File S2 (Table S2).pdf]

Table S2. Key ongoing clinical trials utilizing immunotherapy in resectable NSCLC.

NCT	N	Setting (Neoadjuvant/ perioperative/ adjuvant)	Immunotherapy agent (+/- chemotherapy)	Brief Description	Estimated date of completion
NCT05577702	121	Neoadjuvant	Tislelizumab +/- Ociperlimab OR Tislelizumab +/- LBL-007 (+/- platinum-based chemotherapy)	Phase 2, Stage II-IIA Outcomes: MPR, pCR, EFS, OS, DFS	23 January 2025
NCT04304248	30	Neoadjuvant	Toripalimab (+ platinum-based chemotherapy)	Phase II, Resectable stage IIIA-IIIB, Outcomes = MPR, pCR, resectability rate, DFS	30 July 2026
NCT06465329	120	Neoadjuvant	Cemiplimab (+ platinum-based chemotherapy) +/- REGN7075	Phase II, Resectable stage II-IIIB, Outcomes = MPR, pCR, ORR, EFS, OS, DFS, TRAEs	21 May 2030
NCT04728724	100	Neoadjuvant	Sintilimab (+ platinum-based chemotherapy)	Phase II, Stage III and AGA-ve, Outcomes = MPR, ORR, DFS, PFS, OS, Safety	31 December 2027
NCT05940532	41	Neoadjuvant	Sugemalimab (+ platinum-based chemotherapy)	Phase II, Stage III, Outcomes = PFS, OS, ORR, AEs, QOL	December 2026
NCT05527808	28	Neoadjuvant	Tislelizumab (+ platinum-based chemotherapy)	Phase II, non-squamous NSCLC, Stage II-IIA EGFR+ve and PD-L1 ≥ 1%, Outcomes = MPR, pCR,, ORR, AEs	30 December 2025
NCT06492421	90	Neoadjuvant	Ipilimumab + pembrolizumab	Phase II, Intra-tumoral double	30 December

			OR ipilimumab + durvalumab (+/- idarubicin +/- bevacizumab)	ICIs (+/- chemo) in resectable stage I-IIIA, Outcomes = MPR, pCR, toxicity, response rate, OS	2035
NCT06 743581	24	Neoadjuvant	Cemiplimab + dupilumab	Phase I/II, Resectable AGA-ve Outcomes = Rate of surgery, tolerability, MPR, pCR, EFS, OS	February 20230
NCT05 800340	30	Neoadjuvant	Toripalimab (+ platinum-based chemotherapy)	Phase II, Stage II-IIIB with rare mutations (RET, MET, ERBB2, Exon 20 insertion), Outcomes = MPR, pCR, EFS, OS, AEs	31 December 2026
NCT05 244213	35	Neoadjuvant	Sintilimab (+ carboplatin and nab-paclitaxel)	Phase II, Resectable stage II-IIIB with EGFR+ve Outcomes = MPR, pCR, PFS, OS, AEs	01 December 2025
NCT04 506242	74	Perioperative	Camrelizumab (+ apatinib NAC)	Phase II, Resectable stage II-IIIB with EGFR-ve Outcomes = MPR, pCR, EFS, ORR, DFS, AEs	30 December 2025
NCT06 987734	25	Perioperative	Sugemalimab (+ platinum-based chemotherapy)	Phase II, Stage II-IIIA NSCLC without AGA, Outcomes = MPR, pCR, EFS	31 August 2028
NCT04 943029	30	Perioperative	Camrelizumab (+ platinum-based chemotherapy)	Phase II, Stage III, Outcomes = Rate of surgery, tolerability, MPR, pCR, PFS, OS, AEs	01 August 2024

NCT06617936	36	Perioperative	Tislelizumab (+ recombinant human endostain + platinum-based chemotherapy)	Phase II,<br>Stage III,<br>Outcomes = Rate of surgery, R0 resection rate, tolerability, ORR, EFS, MPR, pCR, PFS, OS, AEs	10 September 2027
NCT04638582	44	Perioperative	Pembrolizumab (+/- platinum-based chemotherapy)	Phase II,<br>Stage IA3-IIA,<br>Outcomes = ctDNA resolution,<br>Imaging response, MPR, pCR, DFS, OS, AEs	27 December 2025
NCT05825625	35	Perioperative	Tiragolumab + atezolizumab (+ platinum-based chemotherapy)	Phase II,<br>Resectable stage II-IIIB,<br>Outcomes = MPR, pCR, EFS, OS, AEs	01 June 2027
NCT06902272	30	Perioperative	Pembrolizumab (+ platinum-based chemotherapy)	Phase II,<br>Resectable stage II-IIIB,<br>ctDNA clearance,<br>pCR, RFS, OS, ctDNA recurrence rate	01 August 2029
NCT06195683	23	Perioperative	Serplulimab monotherapy	Phase II,<br>Stage IB-IIIA with TPS ≥ 50%,<br>Outcomes = pCR, MPR, ORR, OS, EFS	20 June 2025
NCT05360979	42	Perioperative	Envafolimab (+ recombinant human endostatin + chemotherapy)	Phase II,<br>Resectable stage II-IIIB,<br>Outcomes = MPR, pCR, EFS, DFS, OS, AEs	15 May 2025
NCT06449313	21	Perioperative	Cemiplimab (+ platinum-based chemotherapy)	Phase II,<br>Stage III,<br>Outcomes = MPR, pCR, DFS, OS, ORR, R0 resection rate	September 2032

NCT06109402	160	Perioperative arm and adjuvant-only arm	Drug: TQB2450 (+ platinum-based chemotherapy)	Phase II,<br>Resectable stage II-IIIB<br>Outcomes = MPR, pCR, EFS, OS, ORR, QOL	01 November 2030
NCT04973293	20	Perioperative	Sintilimab (+ bevacizumab + platinum-based chemotherapy)	Phase N/A<br>Stage II-IIIA non-squamous NSCLC,<br>Outcomes = safety, feasibility, MPR, imaging response	31 December 2025
NCT04326153	30	Perioperative	Sintilimab (+ platinum-based chemotherapy)	Phase II,<br>Resectable stage IIIA-IIIB,<br>Outcomes = MPR, pCR, DFS, OS, ORR, TRAEs	December 2027
NCT06109402	160	Perioperative arm and adjuvant arm	TQB2450 (+ platinum-based chemotherapy)	Phase II,<br>Resectable stage II-IIIB,<br>Outcomes = MPR, pCR, ORR, EFS, OS, AEs	01 November 2030
NCT06931717	390	Adjuvant	Cemiplimab (vs. observation)	Phase III,<br>Randomized resected stage II-IIIA with PD-L1 ≥1% to adjuvant ICI vs. observation.<br>Outcomes = DFS, OS, AEs	March 2029
NCT06498635	306	Adjuvant	Durvalumab (vs. observation)	Phase III,<br>Randomized resected stage II-IIIB with a pCR to adjuvant ICI vs. observation,<br>Outcomes = DFS, OS, EFS, AEs	15 July 2039

AGA: Actionable genomic alterations, ICI: Immune checkpoint inhibitors, AEs: Adverse events, MPR: Major pathological response, pCR: Pathological complete response, DFS: Disease-free survival, OS: Overall survival, EFS: Event-free survival, ORR: Objective response rate, PD-L1: Programmed death ligand 1, EGFR: Epidermal growth factor receptor.
