# Supplementary material for: A Canadian Perspective on Perioperative Systemic Therapy in Resectable Non-Small Cell Lung Cancer
Source: Curr Oncol. 2025 Dec 30;33(1):20. doi: 10.3390/curroncol33010020 (PMC12840373; doi:10.3390/curroncol33010020)
Supplement: Supplementary file 1 [file curroncol-33-00020-s001.zip › Supplementary File S3 (Table S3).pdf]

Table S3. Summary of the phase II and III trials analyzing radiation for resectable NSCLC.

STUDY	STUDY DESIGN	KEY FINDINGS	SUMMARY
Neoadjuvant Radiation for Early-Stage NSCLC			
MISSILE<br><br>Palma et al [71]<br>Tan et al [72]	• Single arm phase II prospective trial for patients with stage T1-2 N0 M0 NSCLC who underwent SBRT, followed by surgery 10 weeks later<br>• N=36 patients proceeded to surgery<br>• Median follow up of 6.6 years after surgery	• pCR 60% and MPR 63%<br>• 5-year OS 66.7% (95% CI, 48.8%-79.5%)<br>• 5-year DFS 58.3% (95% CI, 40.7%-72.4%)<br>• 6 patients (16.7%) experienced grade ≥3 adverse events, and no grade 5 events	• SBRT and surgery combined is a safe approach, with long-term outcomes similar to those reported with surgery alone
Altorki et al [73]	• Single-centre, open-label, randomized phase II trial comparing neoadjuvant durvalumab alone versus durvalumab plus SBRT (24 Gy in 3 fx), followed by surgery for patients with potentially resectable early-stage NSCLC<br>• N=60 patients enrolled and randomized (30 in each group)	• 26 (87%) of patients in each group underwent surgical resection<br>• MPR in 2 patients (6.7%) in the durvalumab monotherapy arm, vs. 16 patients (53.3%) in the durvalumab + SBRT arm ($p<0.0001$)<br>• 8 of the 16 (50%) patients in the dual therapy group had a complete pathologic response<br>• Grade 3-4 toxicity in 5 (17%) in the monotherapy	• Neoadjuvant durvalumab combined with SBRT is a safe and well tolerated approach with a significantly higher MPR rate compared to neoadjuvant durvalumab alone

		group vs. 6 (20%) in the dual therapy arm, with most frequent event being hyponatremia.	
Neoadjuvant Radiation for Locally Advanced NSCLC (Non-Pancoast)			
National Cancer Institute numbers R9309, INT0139<br><br>Albain et al [77]	• Phase III RCT evaluating induction chemoradiation followed by surgery (if no progression on induction) vs. definitive chemoradiation for resectable stage T1-3 N2 NSCLC	• Improved median PFS in induction arm of 12.8 vs. 10.5 months (p=0.017); 5-year 22.5% vs. 11.1%.<br>• No difference in median OS (23.6 vs. 22.2 months, p=0.24)<br>• An exploratory analysis which excluded pneumonectomy patients from arm 1, showed an OS advantage for lobectomy compared to the chemoradiation arm (median survival 33.6 vs. 21.7 months, p=0.002).	• No significant survival advantage with induction chemoradiation followed by surgery compared to definitive chemoradiation, despite improved PFS.<br>• Chemoradiation followed by resection with lobectomy may represent an option for patients with resectable N2 NSCLC based on exploratory analysis.
Pless et al [78]	• Phase III multicentre RCT investigating the addition of neoadjuvant radiation to chemotherapy in resectable stage IIIA/N2 NSCLC	• No difference in median EFS (11.6 in chemotherapy group vs. 12.8 months in chemoradiation group, p=0.67) between the two groups	• Addition of induction radiation to chemotherapy did not add any benefit, suggesting that one definitive local treatment modality

	• N=232 patients randomized	• No significant differences between the groups in OS	combined with neoadjuvant chemotherapy is adequate.
Thomas et al [79]	• Phase III multicentre trial randomizing patients with stage IIIA-IIIB NSCLC to the following 2 arms:<br>• Control: neoadjuvant chemotherapy, followed by surgery followed by radiation<br>• Intervention: Neoadjuvant chemotherapy followed by radiation followed by surgical resection, with positive margins or unresectable disease offered further radiation<br>• N=558 patients randomized and 524 eligible	• 54% in intervention group and 59% in control group underwent surgery<br>• 37% in interventional group and 32% in control group had complete resection<br>• Mediastinal downstaging and pathological response favoured the intervention group<br>• In pneumonectomy patients, increased treatment-related mortality in the intervention group compared to control (14% vs. 6%)<br>• No difference in PFS	• For resectable stage III NSCLC, neoadjuvant chemoradiation in addition to chemotherapy improves mediastinal downstaging and pathological response, without a survival benefit.<br>• Pneumonectomy should be avoided in patients who underwent induction chemoradiation given increased treatment-related mortality in these patients.
ESPATUE<br><br>Eberhardt et al [80]	• Phase III trial which randomized patients with stage IIIA N2 NSCLC after induction sequential chemotherapy followed by chemoradiation (45 Gy), to either definitive chemoradiation	• No significant differences in 5-year OS (40% vs. 44%, p=0.34) and PFS (35% vs. 32%, p=0.75).	• Both strategies achieved excellent 5-year OS and PFS, which were not statistically different.<br>• Trial closed early due to slow accrual and end of funding, therefore

	boost to 65-71 Gy or surgery.<br>• N=246 of 500 planned patients		underpowered to meet its primary end points.
Radiation Therapy Oncology Group protocol 0229<br><br>Suntharalingam et al [81]	• Phase II multi-institutional trial for patients with stage III NSCLC with pathologically proven N2/N3 disease<br>• Induction chemotherapy followed by concurrent radiation (50.4 Gy to mediastinum and primary tumor with boost of 10.8 Gy to all gross disease)<br>• Mediastinum was pathologically reassessed after chemoradiation	• 43 patients (75%) were evaluated for primary endpoint of MNC<br>• 37 patients underwent resection<br>• 27 patients (63%) achieved MNC<br>• 14% incidence of grade 3 post-operative lung complications<br>• 2-year OS was 54%, but 75% for those who achieved nodal clearance, 52% for those with residual nodal disease and 23% for those who were not evaluable for the primary endpoint (p=0.0002)<br>• 2-year PFS was 33%	• Neoadjuvant chemoradiation can sterilize mediastinal nodal disease.
SQUAT trial (WJOG 12119L)<br><br>Hamada et al [83]	• Phase II multi-centre trial of patients with stage IIIA to IIIB NSCLC with N2 disease.<br>• Patients underwent concurrent chemoradiation and durvalumab, followed by surgery within 2-6	• MPR 63% (90% CI 47-78%) and pCR 23%<br>• 2-year PFS 43%<br>• 2-year OS 76%<br>• 48% rate of grade 3-4 adverse events, including one treatment-related mortality	• Higher MPR rate compared to prior studies, but no OS improvement

	weeks, followed by adjuvant durvalumab.<br>• N=31 patients enrolled		
Neoadjuvant Radiation for Resectable Superior Sulcus Tumors			
Southwest Oncology Group Trial 9416 (Intergroup Trial 0160)<br><br>Rusch et al [88]	• Phase II trial of patients with T3-4 N0-1 superior sulcus NSCLC who received induction chemoradiation (radiation of 45 Gy), followed by thoracotomy for patients with stable or responding disease.<br>• N=110 patients recruited, with induction completed by 104 (95%) and 88 of 95 (80%) eligible patients undergoing surgery.	• 83 patients had complete resection<br>• 2 died postoperatively<br>• pCR or minimal microscopic disease in 61 (56%) of resection specimens<br>• 5-year survival 44% for all patients and 54% after complete resection<br>• No difference between T3 and T4 tumors seen<br>• Disease progression mostly distantly	• Combined-modality approach with induction chemoradiation and surgery is feasible and associated with high rates of complete resection and pCR<br>• pCR associated with improved survival than when residual disease present (p=0.02)
Japan Clinical Oncology Group Trial 9806<br><br>Kunitoh et al [89]	• Phase II trial of superior sulcus NSCLC evaluating trimodality therapy consisting of chemoradiation (radiation of 45 Gy in 25 fx with a 1-week split course) followed by surgery	• Pathologic complete resection in 51 patients (68%), with 12 patients with pathologic complete response<br>• 3 treatment-related deaths<br>• 3-year DFS was 49% and 5-year DFS 45%	• Trimodality therapy is a safe and effective treatment for patients with superior sulcus tumor

	• N=76 patients enrolled, 75 of which were assessable. 57 patients underwent surgical resection.	• 3-year OS was 61% and 5-year OS 56%	
Adjuvant Radiation for Resectable NSCLC			
PORT-C<br><br>Hui et al [98]	• Phase III randomized trial of patients with pIIIA-N2 NSCLC treated with complete resection and 4 cycles of chemotherapy<br>• Patients randomized into PORT of 50 Gy (n=202, but 184 in final analysis) or observation (n=192, but 180 in final analysis)<br>• Median follow up of 46 months	• 3-year DFS 40.5% with PORT vs. 32.7% with observation (median, 22.1 vs. 18.6 months). Non-significant without adjustment (hazard ratio [HR], 0.84; 95% CI, 0.65-1.09; *P* = .20). Significant on exploratory analysis stratified by lymph node burden (HR, 0.75; log-rank *P* = .04)<br>• 3-year OS 78.3% vs. 82.8% (HR, 1.02; *P* = .93)<br>• No radiation grade 4 or 5 toxicities	• PORT did not demonstrate a DFS or OS benefit
Lung ART, IFCT 0503<br><br>Le Pechoux et al [99]	• Phase III randomized, superiority trial comparing PORT of 54 Gy to observation in stage IIIA patients with complete resection, nodal exploration and	• 3-year DFS 47% with PORT vs. 44% with observation<br>• Median DFS 30.5 months with PORT and 22.8 months with observation (hazard ratio 0.86; 95% CI 0·68–1.08; p=0·18)	• PORT was not associated with an increased DFS and therefore cannot be recommended as standard of care in patients with stage IIIA N2 NSCLC

	pathologically proven N2 disease<br>• Patients predominantly staged using ^{18}F-FDG PET-CT<br>• Patients received perioperative chemotherapy<br>• 3D conformal PORT predominantly used<br>• N=501 patients randomized (252 to PORT and 249 to no PORT)<br>• Median follow up of 4.8 years	• 26 patients (11%) with late grade 3-4 cardiopulmonary toxicity in PORT group (most commonly pneumonitis in 5%), versus 12 (5%) in observation group<br>• 3 treatment-related deaths, all of which were in the PORT group	

SBRT: stereotactic body radiation therapy, MPR: Major pathological response, pCR: Pathological complete response, HR: Hazard ratio, CI: Confidence interval, OR: Odds ratio, PFS: Progression-free survival, OS: Overall survival, DFS: Disease-free survival, ORR: Objective response rate, PORT: postoperative radiotherapy
