# Supplementary material for: A Canadian Perspective on Perioperative Systemic Therapy in Resectable Non-Small Cell Lung Cancer
Source: Curr Oncol. 2025 Dec 30;33(1):20. doi: 10.3390/curroncol33010020 (PMC12840373; doi:10.3390/curroncol33010020)
Supplement: Supplementary file 1 [file curroncol-33-00020-s001.zip › Supplementary File S4 (Table S4).pdf]

Table S4. Key ongoing clinical trials utilizing radiation in resectable NSCLC.

NCT	Setting (Neoadjuvant/ Adjuvant)	Brief Description	Estimated date of completion
06877299	Neoadjuvant	Phase II, n=30, stage IIa-IIIb NSCLC<br><br>Low-Dose Radiation (2Gy*2d) + SBRT (10Gy*3d) ◎ PD-1 inhibitor (Tislelizumab) + platinum-based chemotherapy ◎surgery<br><br>Outcomes: pCR (primary), MPR, R0-resection rate, EFS	01 January 2028
06718309	Neoadjuvant	Phase II, n=40, stage II-IIIa, IIIB (T3-4N2, T4 only >7cm) NSCLC, EGFR/ALK wild-type<br><br>Chemoimmunotherapy (1 cycle) , SBRT ◎ , Chemoimmunotherapy x 2 cycles ◎ , surgery.	30 April 2029
04245514	Neoadjuvant	Phase II, n=90, stage T1-3 N2, T4 (only if due to size>7cm) N2, NSCLC<br><br>Chemotherapy x3 cycles (cisplatin + docetaxel) ◎ Immune-modulated radiotherapy (random assignment between 20x2 Gy week daily, 5x5 Gy week daily, 3x8 Gy q2d with concurrent immunotherapy) ◎surgery ◎ PORT if indicated ◎ adjuvant durvalumab<br><br>Outcomes: 12-month EFS (primary endpoint), EFS, RFS after R0 resection, OS, OR (objective response) after neoadjuvant chemotherapy, OR after neoadjuvant immuno-radiotherapy, pCR, MPR, overall MPR, Nodal down-staging to < pN2, complete resection	01 December 2031
05798845	Neoadjuvant	Phase II, n=124, stage II-IIIA (N+) NSCLC<br><br>Arm 1: SBRT (24 Gy/3 fx, d1-3) + LDRT (2Gy/2Fx, d1-2, d22-23) + Toripalimab ◎ surgery<br>Arm 2: Chemotherapy + Toripalimab ◎surgery	31 December 2026

		Outcomes: pCR (primary), MPR, perioperative complications, completion of surgery, R0 resection rate, treatment-emergent adverse event, EFS, OS, circulating tumor DNA, immune subtypes, PD-L1 expression and tumor mutation burden.	
06714708	Neoadjuvant	Phase II, n=86, stage IIb-III, N2 NSCLC<br><br>Low dose radiation (30 Gy/15 fx) + sintilimab + chemotherapy ◎ surgery<br><br>Outcomes: pCR, MPR, ORR, EFS	01 June 2027
04202809	Neoadjuvant	Phase II, n=90, stage IIIa-IIIb<br><br>Arm 1: Neoadjuvant Chemotherapy + Chemoradiation + Durvalumab ◎ surgery<br>Arm 2: Neoadjuvant Chemotherapy + Chemoradiation ◎ surgery<br><br>Outcomes: PFS (primary), OS, 2-year OS rate, Functional response, RECIST response, RECIST criteria, EORTC QLQ-C30, EORTC QLQ-LC13, FACT-L	01 April 2025
07050056	Neoadjuvant	Phase II, single arm, n=20, stage<br><br>Tislelizumab (3-4 cycles) + Radiation (IMRT 40 Gy/20 fx) ◎ surgery<br><br>Outcomes: pCR (primary), MPR rate, 1-year EFS rate	31 December 2027
03694236	Neoadjuvant	Phase II single arm, n=39, stage III (including N2)<br><br>Radiation (45 Gy/25 fx) + paclitaxel + carboplatin + durvalumab ◎ surgery<br><br>Outcomes: pCR (primary), objective response rate (ORR), DFS, OS, clinical and pathologic downstaging rate	01 May 2027
06800339	Neoadjuvant	Phase I, single arm, n=18, stage II-IIIC (N3) NSCLC	01 March 2030

		Nivolumab + Chemotherapy (carboplatin or cisplatin + pemetrexed, paclitaxel or gemcitabine) + SBRT ◎ surgery<br><br>Outcomes: tolerability of adding sub-ablative, immunosensitizing radiation to SOC neoadjuvant chemoimmunotherapy (primary), adverse events, pCR, MPR, and measure of definitive resection	
06598527	Neoadjuvant	Phase III, randomized, multicenter, prospective study<br>Stage IIa-IIIa, IIIb (T3-T4 N2) NSCLC<br><br>Arm 1: SBRT (24 Gy/3 fx) + Tislelizumab + Chemotherapy (platinum-based double-agent chemotherapy ◎ surgery<br>Arm 2: Tislelizumab + Chemotherapy (platinum-based double-agent chemotherapy ◎ surgery<br><br>Outcomes: EFS (primary), MPR, PCR, R0 resection rates, OS, safety	30 January 2030
05319574	Neoadjuvant	Phase II, n=46, stage II-III NSCLC<br><br>SBRT (8 Gyx3 days) ◎ Tislelizumab + concurrent platinum-based doublet chemotherapy ◎ surgery<br><br>Outcomes: MPR (primary), pCR, Resected Rate, DFS	31 May 2026
05500092	Neoadjuvant	Phase II, randomized, n=52, stage IIA-IIIB NSCLC<br><br>Arm 1: Nivolumab + platinum-doublet chemotherapy ◎ surgery<br>Arm 2: Nivolumab + platinum-doublet chemotherapy + sub-ablative SBRT (8 Gy x3) ◎ surgery<br><br>Outcomes: CPR (primary), MPR, EFS	01 July 2025
04933903	Neoadjuvant	Phase II, single arm, n=25, stage IB-IIIB (T2-3N0, T1-T3N1-2) NSCLC	01 December 2025

		Ipilimumab + nivolumab + SBRT (7Gy x 1; 4Gy x 2) ◎ surgery<br><br>Outcomes: Number of patients with pathologic response (primary), incidence of treatment-emergent adverse events (safety & tolerability)	
06878274	Adjuvant	Phase II, n=80, stage T1-4 N2-4 NSCLC<br><br>Neoadjuvant chemoimmunotherapy ◎ surgery ◎ PORT for residual disease<br><br>Outcomes: DFS (primary), OS, grade 3 toxicity, locoregional control, DMFS	01 February 2030
06008730	Adjuvant	Phase II, n=20, Resected N2 NSCLC<br><br>Surgery ◎ Proton beam radiation therapy<br><br>Outcomes: Incidence of adverse events (primary), radiation dose to the immune compartment, DFS, OS	31 December 2027

SBRT: stereotactic body radiation therapy, MPR: Major pathological response, pCR: Pathological complete response, HR: Hazard ratio, CI: Confidence interval, OR: Odds ratio, PFS: Progression-free survival, OS: Overall survival, DFS: Disease-free survival, ORR: Objective response rate, EGFR: Epidermal growth factor receptor, ALK: Anaplastic lymphoma kinase.
